# Changing trends in anti-psychotic prescription pattern in Pakistan

**DOI:** 10.12669/pjms.35.3.136

**Published:** 2019

**Authors:** Mazhar Malik, M. Usman Ghani, Wardha Mazhar, Nargis Munir

**Affiliations:** 1 *Prof. Dr. Mazhar Malik, Professor of Psychiatry, HoD Department, Department of Psychiatry and Behavioural Sciences, Rawal Institute of Health Sciences, Rawal General and Dental Hospital, Lehtrar Road, Islamabad, Pakistan*; 2 *Dr. M. Usman Ghani, Assistant Professor of Psychiatry, Department of Psychiatry and Behavioural Sciences, Rawal Institute of Health Sciences, Rawal General and Dental Hospital, Lehtrar Road, Islamabad, Pakistan*; 3 *Dr. Wardha Mazhar, Assistant Professor of Pharmacology, FUMC, Islamabad, Pakistan*; 4 *Nargis Munir, Clinical Psychologist, Department of Psychiatry and Behavioural Sciences, Rawal Institute of Health Sciences, Rawal General and Dental Hospital, Lehtrar Road, Islamabad, Pakistan*

**Keywords:** Polypharmacy, Prescription pattern, Psychotropic drugs, Schizophrenia. Antipsychotics

## Abstract

**Background and Objective:**

This study was designed to identify the changing trends in Anti-psychotic prescription pattern in Pakistan. It was part of the research project Research on East Asian Psychotropic Prescription Pattern (REAP) carried out to identify the prescription patterns of schizophrenic patients in different countries located in Asia.

Our objective was to assess the trend and change of psychotropic drug prescriptions for patients with schizophrenia.

**Methods:**

The design of the study was quantitative and of descriptive epidemiology. This study was carried out from 30^th^ March 2017. Data was collected on a unified protocol by the Psychiatrists from Pakistan. Three (3) centers i.e., Lahore, Karachi and Islamabad provided the data. Indoor and outdoor cases with Schizophrenia were recruited. A web based recording system for collection of data done at Taipei Taiwan, and statistical analysis was performed and transferred to all participating centers including Pakistan.

**Results:**

The main findings of the study were that majority of the patients were prescribed antipsychotic poly pharmacy drug. It was also found that Anxiolytics, anti-depressants and Anti-parkinsonian drugs were also co-prescribed.

**Conclusion:**

It was concluded that antipsychotic poly pharmacy along with Anxiolytics, anti-depressants and Anti-parkinsonian drugs were prescribed to patients with schizophrenia in Pakistan.

## INTRODUCTION

International Collaborative Studies on Psychotropic Prescription, a Research on East Asian Psychotropic Prescription Pattern (REAP) is the largest and the longest international collaborative research project in Asia. REAP undertook large scale surveys on prescription patterns of schizophrenic patients in different countries located in Asia. This research was initially supported by Japan Society for Promotion of Sciences (JSPS).

REAP has conducted four survey studies in Asia. The first survey was carried out in July 2001 in China, Hong Kong, Indonesia, Japan, Korea, Singapore, Malaysia and Taiwan. The objective of this study was to find out the class of antipsychotic drugs used for schizophrenic in patients. The survey in 2001 reported the dominant use of the First-Generation Antipsychotics (FGAs) such as Chlorpromazine and Haloperidol over the Second-Generation Antipsychotics (SGAs) such as Quetiapine and Risperidone. To review the change since 2001, the second survey was carried out in July 2004 in mainland China, Hong Kong, Korea, Japan, Taiwan and Singapore that followed the same research protocol and questionnaire. The findings of follow-up survey suggested that the use of SGA’s increased sharply in these countries since 2001.^1^

The fact that anti-depressants prescribed widely to non depressants patients were taken into consideration and the first REAP survey on antidepressant was carried out in March 2004, and studied medical records of patients receiving antidepressants. The results of this survey suggested that one third of patient receiving antidepressants had other diagnosis such as somatoform disorders as well as schizophrenia.^2^

The third survey was carried out in 2008 in China, Korea, Japan, Taiwan, Hong Kong, Singapore, Thailand, Malaysia and India. This survey was also conducted on the use of antipsychotic drugs. With the participation of India, Malaysia and Thailand, REAP became the research group covering almost all the countries of Asia. This survey showed that SGA’s are the main antipsychotic drugs for the treatment of Schizophrenia in Asia.^3^ Another survey on the use of antidepressants was conducted in 2014 by East Asia and South Asia.^4^,^5^

Possible impacts of REAP were for the better understanding and improvement of prescription of antipsychotics in participating countries and contribution to scientific exchange among Asian psychiatrists. It has influenced the prescription pattern in participating countries.

The standard treatments for Schizophrenia during the last 60 years have been First Generation Antipsychotic drugs. The First Generation Antipsychotic drugs (classical or typical) showed a great efficacy at reducing positive symptoms like auditory hallucinations and delusions in Schizophrenia.

Various researches were carried out on Asian Psychotropic Prescription Pattern (REAP) to access antipsychotic medications and psychotropic co-treatments in Asian patients with schizophrenia.^6^ One of the studies was on the use of Clozapine in patients with Schizophrenia. The result of this study showed that the use of Clozapine prescription has considerably decreased over time from 2001 to 2009 in China, while its use has increased in Korea and Singapore during this period.^7^ Another REAP study was conducted on the prescribing pattern of antipsychotic medications in patients with Schizophrenia in a tertiary care hospital in India. The results showed that 6% patients were on monotherapy; whereas Asenapine was the only drug used while rests of 94% patients were on combination therapy.^8^ Another study was conducted on the cardiovascular risks of atypical antipsychotic drug treatment in 2007. According to this study, typical antipsychotics are the drug of choice for patients with Schizophrenia. Atypical antipsychotics are much better than the conventional antipsychotic and do not cause extra pyramidal symptoms. Another study was conducted on the use of antipsychotic polypharmacy in patients with Schizophrenia. The major findings of this study were that multiple antipsychotics are widely prescribed to more than one third of the schizophrenic patients in Asian region. In addition, Antipsychotic PolyPharmacy (APP) prescription pattern decreased in Asia from 46.9% in 2001 to 38.3% in 2004, but then increased to 42.9% in 2009. The frequency of Antipsychotic Poly Pharmacy in the current study is in the range of previous findings.^9^ The objective of current study was to assess the trend and change of psychotropic drug prescriptions for patients with schizophrenia.

## METHODS

### Research Design

The design of the study was quantitative and of descriptive epidemiology. Data was collected on a unified protocol by the Psychiatrists of the participating countries from Pakistan. The data collection for the current study was started from 30^th^ March 2017. Initially Five (5) centers were selected for data collection, while three (3) centers i.e., Lahore, Karachi and Islamabad provided the data. Indoor and outdoor cases with Schizophrenia were recruited. The prescribing patterns of psychiatrists were analyzed, taking consideration of the available psychotropic drugs on the market and different geographical and practicing systems. The data was collected on the short-form version of the questionnaire. Demographic details like age, gender, duration of illness, rural urban setting, were taken before data collection.

### Inclusion criteria

1. Diagnosed patients with Schizophrenia according to Diagnostic and Statistical Manual 5 (DSM 5) or International Classification of Diseases (ICD- 10).
2. Being a patient on the day of survey.
3. Willingness to participate in the study.

### Exclusion criteria

1. The patients who were not willing to participate were excluded.
2. Presence of serious illness, for example serious cardiovascular illness, recent history of stroke, hepatic failure and renal failure.

### Data Collection and Analysis

Ethical permission was taken from the administration of the Rawal Institute of Health Sciences Islamabad Pakistan, before collecting the data. Data was collected by the psychiatrist in front of patient.

Informed consent was taken from the patients for participating in the REAP survey before the collection of data. The data was collected through the questionnaire which includes demographic details, psychiatric symptoms, psychotropic medications, co morbidities, physical conditions, and adverse drug reactions, considering different geographical and practicing systems and available psychotropic drugs in the market. Diagnosed cases of schizophrenia were the population of the study and all those patients meeting the criteria were the sample of study. Nonrandom, purposive sampling technique was used. The questionnaire used for data collection was a reliable and validated tool for the patients of schizophrenia. Duration of acute and chronic illness was taken while collecting data.

This is a web based recording system for collection of data done at Taipei Taiwan, and statistical analysis was performed and transferred to all participating centers including Pakistan.

## RESULTS

The demographic details from three centers of Pakistan are given in Table-I. The demographic characteristics included age, gender, treatment setting, and acute and chronic cases. Table-II shows percentage of patients on typical, atypical and on long acting antipsychotics. (Table-III shows co prescription of other drugs along with the antipsychotics while Table-IV shows top ten antipsychotics and co prescription of other drugs.

**Table-I:** Demographic details of patients.

Variables	Total N=298	Centre Case 246 (Lahore)	Centre Case 13 (Karachi)	Centre Case 39 (Islamabad)
***Age (20-65years)***				
Mean= SD	36.9	36.6	41.2	37.0
***Gender n (%)***				
Male	168 (56.4%)	138 (56%)	8 (61.5%)	22 (56.4%)
Female	130 (43.6%)	108 (43.9%)	5 (38.5%)	17 (43.6%)
***Treatment setting n (%)***				
Outpatient	156 (52.3%)	108 (43.9%)	11 (84.6%)	37 (94.9%)
Inpatient	142 (47.7%)	138 (56.1%)	2 (15.4%)	2 (5.1%)
Acute cases	49 (31.4%)	46 (42.6%)		3 (8.1%)
Chronic cases	12 (37.5%)	11 (55.0%)		1 (100.0%)

**Table-II:** Typical and Atypical Antipsychotics.

Class of Drugs	Drug Name	N=298	%
***Typical Anti-psychotic drugs***			
	Haloperidol	79	26.5%
	Trifluoperazine	17	5.7%
	Clopenthixol	1	0.3%
	Chlorpromazine	1	0.3%
***Atypical Anti-psychotic drugs***			
	Risperidone	186	62.4%
	Quetiapine	55	18.5%
	Clozapine	33	11.1%
	Aripiprazole	18	6.0%
	Olanzapine	18	6.0%
	Amisulpride	2	0.7%
	Ziprasidone	2	0.7%
Poly pharmacy(prescription of more than one antipsychotic drug)	Typical and a typical antipsychotics	151	51.7%
Long acting-antipsychotic	Fluphenazine decanoate	38	12.8%
	Zuclopenthixol decanoate	20	6.7%
	Flupentixol decanoate	8	2.7%
	Haloperidol decanoate	8	2.7%

**Table-III:** Co-prescription of other medications.

Mood-Stabilizer			
	Valproic acid	58	19.5%
	Carbamazepine	15	5.0%
	Lamotrigine	6	2.0%
	Lithium	2	0.7%
	Oxcarbazepine	2	0.7%
Anxiolytic	Clonazepam	116	38.9%
	Lorazepam	36	12.1%
	Oxazepam	10	3.4%
	Alprazolam	8	2.7%
	Bromazepam	6	2.0%
Hypnotics used		21	7%
Anti-depressants used		56	18.8%
Anti parkinsonian drug used		207	65.9%

**Table-IV:** List of Top ten Antipsychotics and commonly prescribed Mood Stabilizers, Anxiolytics and Anti Parkinsonian Drugs.

Antipsyhotics (Top ten used)	Drug Name	N	%
Other drugs(Top ten used)	Risperidone	186	62.4%
	Haloperidol	79	26.5%
	Quetiapine	55	18.5%
	Fluphenazine decanoate	38	12.8%
	Clozapine	33	11.1%
	Zulcopenthixol decanoate	20	6.7%
	Aripiprazole	18	6.0%
	Olanzapine	18	6.0%
	Trifluoperazine	17	5.7%
	Antiparkinsonian drugs	207	65.9%
	Anxiolytic(Clonazepam)	116	38.9%
	Mood Stabiliser(Valproic Acid)	58	19.5%
	Antidepressants	56	18.8%
	Anxiolytic(Lorazepm)	36	12.1%
	Hypnotics	21	7%
	Mood Stabilisers(Carbamezipine)	15	5.0%

## DISCUSSION

Schizophrenia is a psychotic illness with a range of clinical symptoms including auditory hallucinations, delusions and anhedonia that coalesce to form a devitalizing syndrome affecting all functional domains. These symptoms respond in different manner to treatment depending on various factors. A study conducted on the pattern of use of antipsychotic drugs found that 31% of the patients received a combination of atypical and typical antipsychotics in greater than maximum recommended doses.^10^

The treatment of Schizophrenia has been challenging despite advancing pharmacological treatment which often leads to the use of two or more antipsychotics to treat refractory and residual psychotic signs and symptoms. A systematic review with a pooling data across four decades from 1979 to 2009 found the median rate for the prescription of antipsychotic polypharmacy to be 19.6% globally and 32% in Asia which was higher as compared to other regions such as North America and Oceania.^11^

Another study on relation of antipsychotic polypharmacy and quality of life in patients with Schizophrenia in China suggested that the rate of APP prescription was 31 with 89% on two antipsychotics and 10.4% received three or more antipsychotics. It also showed patients prescribed on APP were mainly on higher doses of SGAs along with anticholinergics.^12^

The current study has been conducted to assess the trends and changes in psychotropic drug prescriptions for patients with Schizophrenia. The findings suggested that the use of typical and typical antipsychotic drugs is 15.3% and 31.6% respectively, whereas the use of polypharmacy is 51.7%. Co-prescription pattern of other drugs showed that 77% mood stabilizers, 56% antidepressants, 57.5% anxiolytics, 7% hypnotics and 65% antiparkinsonian drugs are being given to the patients on top with antipsychotics. These findings regarding polypharmacy are in line with a similar study conducted in 2014 in a developing country which showed 31% of patients were receiving a combination of typical and atypical antipsychotics.^13^

Another study conducted in Pakistan suggested that the pharmacotherapy of patients consisted mainly of antipsychotic drugs i.e.; 86%. Other drugs that were prescribed included anxiolytics, antiparkinsonian drugs, anticonvulsants and antidepressants. The results regarding antipsychotic drug use are consistent with our study showing 50% of patients taking typical antipsychotics and 56% on atypical antipsychotics.^14^

### Study Limitations

Data has been taken from three centres of Pakistani, Karachi, Lahore, and Islamabad only. Data details from other centers were not provided.

## CONCLUSION

It was concluded that typical and atypical antipsychotic poly pharmacy were prescribed to patients with schizophrenia in Pakistan. It was also concluded that mood stabilizers, antidepressants, anxiolytics, hypnotics and antiparkinsonian drugs are being given to the patients on top with antipsychotics.
